# Association between latent profiles of disease risk perception and health-promoting behaviors among adult offspring of patients with type 2 diabetes mellitus: a cross-sectional study

**DOI:** 10.3389/fpubh.2026.1928062

**Published:** 2026-10-23

**Authors:** Fan Wang, Songmei Cao, Qiaoyan Liu, Houjuan Zu, San Fan, Jingjing Wang

**Affiliations:** 1 Department of Endocrinology and Metabolism, Affiliated Hospital of Jiangsu University, Zhenjiang, Jiangsu, China; 2 Department of Nursing, Affiliated Hospital of Jiangsu University, Zhenjiang, Jiangsu, China

**Keywords:** disease risk perception, health behavior, latent profile, offspring, type 2 diabetes mellitus

## Abstract

**Objective:**

To identify latent profiles of disease risk perception among adult offspring of patients with type 2 diabetes mellitus and examine their associations with health-promoting behaviors.

**Methods:**

A quantitative, analytical cross-sectional study was conducted using convenience sampling. Data were collected from August 2025 to January 2026 from adult offspring of patients with type 2 diabetes mellitus who accompanied their parents to the outpatient or inpatient departments of Endocrinology and Metabolism at a tertiary Grade A hospital in Zhenjiang, Jiangsu Province, China. Participants completed a general information questionnaire, the Tripartite Model of Risk Perception (TRIRISK), and the Health-Promoting Lifestyle Profile II Revised (HPLP-II R). Latent profile analysis was performed to identify distinct profiles of disease risk perception. Independent-samples *t* tests or one-way analysis of variance were used for univariate comparisons, and multiple linear regression analysis was performed to examine the associations between disease risk perception profiles and health-promoting behaviors.

**Results:**

A total of 301 offspring of patients with type 2 diabetes mellitus completed the survey. Their disease risk perception was classified into three latent profiles: the low risk perception profile, the affective-low risk perception profile, and the affective-elevated risk perception profile. Multiple linear regression analysis showed that monthly per capita household income and disease risk perception profiles were significantly associated with health-promoting behaviors among offspring of patients with type 2 diabetes mellitus (*p* < 0.05).

**Conclusion:**

Disease risk perception among adult offspring of patients with type 2 diabetes mellitus is heterogeneous, and different risk-perception profiles are independently associated with health-promoting behaviors. Healthcare professionals can prioritize assessing the disease risk perception profile of the offspring, so as to provide a theoretical basis and practical guidance for formulating prevention strategies targeting disease risk perception to improve health behaviors among offspring of patients with type 2 diabetes mellitus.

## 1 Introduction

The global diabetes map released by the International Diabetes Federation (IDF) shows that in 2024, China had the largest number of diabetes patients aged 20–79 (148 million), and it is projected that this number will increase to 168.3 million by 2050 (1). Type 2 diabetes constitutes approximately 90% of diabetes cases in China (2), and presents a notable risk of intergenerational inheritance (3). Evidence suggests that offspring have a 15% risk of developing type 2 diabetes with one affected parent (4), whereas the risk rises to 75% if both parents are diagnosed with the disease, which is 2–4 times higher than that of descendants with nondiabetic parents (5). Diabetes is not only a personal health crisis, but also a global socio-economic crisis. Its severity and scope make it a pressing public health priority. The IDF calls for investment in preventive medical programs, so as to identify high-risk individuals at an early stage and help delay or prevent the occurrence of diabetes and its related complications (1). The high prevalence rate among the offspring of type 2 diabetes patients is not only related to genetic factors, but also to the unhealthy lifestyle of the family (6). Risk perception refers to an individual’s subjective feelings and judgments regarding susceptibility to disease and may influence health behaviors and self-management (7). Previous studies concerning risk perception in the field of diabetes have mostly adopted cross-sectional designs to explore risk perception among diagnosed patients or high-risk populations, yet individual heterogeneity has been largely overlooked (8–10). Furthermore, it remains unclear whether differences exist in health behaviors among offspring of patients with type 2 diabetes stratified by distinct levels of disease risk perception. Latent profile analysis (LPA) (11) enables the classification of populations with distinct latent characteristics and maximizes intergroup differences in these attributes. Therefore, this study adopted LPA to identify latent profiles of disease risk perception among offspring of patients with type 2 diabetes, and to explore the associations between these latent profiles and health-promoting behaviors. The findings will provide a theoretical basis for developing prevention strategies targeting disease risk perception, fostering sound health behaviors in this population, and halting the natural progression of type 2 diabetes.

## 2 Methods

### 2.1 Study design

This study employed a quantitative, analytical cross-sectional design, with data collected through a face-to-face questionnaire survey.

### 2.2 Participants and sampling

Using the convenience sampling method, from August 2025 to January 2026, 301 offspring accompanying patients with type 2 diabetes in the outpatient and inpatient departments of the Endocrinology and Metabolism Department of a tertiary grade A hospital in Zhenjiang City, Jiangsu Province, were selected as the survey subjects. Inclusion criteria: ① At least one of the parents has been diagnosed with type 2 diabetes (12); ② Age≥18 years old; ③ the consciousness is clear and communication is normal. Exclusion criteria: ① People who have been diagnosed with diabetes; ② Taking into account other diseases affected by the study, such as malignant tumors and the use of glucocorticoids, etc. The sample size was estimated according to the recommendation that the number of participants in multivariable studies should be approximately 10 times the number of independent variables. Fourteen candidate independent variables were considered; therefore, at least 140 valid participants were required. Allowing for an anticipated 20% rate of invalid or incomplete questionnaires, the minimum recruitment sample size was calculated as 140/(1–0.20) = 175 participants. Ultimately, 301 valid questionnaires were included in the analysis (13).

### 2.3 Measures

#### 2.3.1 General information questionnaire

The research team conducted a literature review and independently designed the questionnaire based on the research objectives, including gender, age, Place of residence, educational level, marital status, employment status, monthly per capita household income, the role of the sick parent, the duration of the sick parent’s diabetes, whether the participant was aware of the genetic risk of diabetes, whether they lived with the sick parent, whether the sick parent had diabetes-related complications, and type of diabetes treatment received by the parent with type 2 diabetes.

#### 2.3.2 The tripartite model of risk perception

The Tripartite Model of Risk Perception (TRIRISK) was developed by Ferrer et al. (14) and translated and culturally adapted into Chinese by Zhou et al. (15). It is used to assess individuals’ perceived risk of developing a chronic disease. It consists of three dimensions: deliberative risk perception, affective risk perception, and experiential risk perception, with a total of 18 items. Each item is scored using the Likert 7-point scale, with a total score ranging from 18 to 126. The higher the score, the greater the individual’s perceived risk of contracting a certain disease. The Chinese version demonstrated good content, construct, and convergent validity. The S-CVI was 0.88, and exploratory factor analysis identified three factors explaining 80.115% of the total variance; confirmatory factor analysis further supported the three-factor structure. The total Cronbach’s *α* coefficient was 0.953, and the split-half reliability coefficient was 0.935. In the present study, the overall Cronbach’s *α* coefficient was 0.958, the split-half reliability coefficient was 0.904, and Cronbach’s *α* coefficients for the three dimensions ranged from 0.871 to 0.945.

#### 2.3.3 Health promotion lifestyle profile-II R (HPLP-II R)

The Health Promotion Lifestyle Scale-II R was revised and adapted by Cao Wenjun et al. (16) based on the Health Promotion Lifestyle Scale-II (HPLP-II). After local adaptation, it is used to assess the health behavior levels of the Chinese population. It consists of 6 dimensions: nutrition (6 items), physical exercise (8 items), health responsibility (11 items), interpersonal relationships (5 items), spiritual growth (5 items), and stress management (5 items). Each item is scored using the Likert 4-point scale, with a total score ranging from 40 to 160. The higher the score, the better the level of health-promoting lifestyle. The Chinese HPLP-II R showed acceptable psychometric properties, with a six-factor structure explaining 48.95% of the total variance and satisfactory convergent and discriminant validity. Cronbach’s *α* coefficients for the six dimensions ranged from 0.63 to 0.81, and the test–retest reliability coefficient was 0.69. In the present study, the overall Cronbach’s *α* coefficient was 0.971.

### 2.4 Data collection

The purpose and significance of the study were explained to eligible participants, and informed consent was obtained before data collection. Trained members of the research team conducted face-to-face, one-on-one questionnaire surveys using standardized instructions. Participants completed the questionnaires independently whenever possible. For participants who were unable to complete the questionnaires independently, researchers recorded their responses without interpretation. Completed questionnaires were collected on site and checked for completeness.

### 2.5 Data analysis

Statistical analyses were performed using IBM SPSS Statistics version 25.0 and Mplus version 8.3. Continuous variables with approximately normal distributions are presented as means and standard deviations (mean ± SD), whereas categorical variables are presented as frequencies and percentages. For univariate analyses of HPLP-II R scores, independent-samples *t*-tests were used for variables with two categories, and one-way analysis of variance (ANOVA) was used for variables with three or more categories. Latent profile analysis (LPA) was performed in Mplus 8.3 using the three dimensions of TRIRISK as manifest indicators. Models with one to four latent profiles were sequentially estimated. Model selection was based on the Akaike information criterion (AIC), Bayesian information criterion (BIC), sample-size-adjusted BIC (aBIC), entropy, Lo–Mendell–Rubin adjusted likelihood ratio test (LMR-LRT), bootstrap likelihood ratio test (BLRT), class size, and interpretability of the profiles. Lower AIC, BIC, and aBIC values indicated better model fit, whereas entropy values closer to 1 indicated greater classification accuracy. Statistically significant LMR-LRT and BLRT results indicated that a k-profile model provided a better fit than a k − 1-profile model. Multiple linear regression analysis was conducted with the total HPLP-II R score as the dependent variable. Variables that showed statistically significant associations with HPLP-II R scores in the univariate analyses, along with the latent disease risk perception profiles, were simultaneously entered into the regression model using the Enter method. Categorical variables were dummy coded using specified reference categories. Regression coefficients (B), standard errors (SE), standardized regression coefficients (*β*), 95% confidence intervals (CIs), *t*-values, and *p*-values were reported. Multicollinearity was assessed using tolerance and variance inflation factor (VIF) values, and the independence of residuals was evaluated using the Durbin–Watson statistic. Standardized residuals were also examined to identify potential outliers. The coefficient of determination (*R*^2^), adjusted *R*^2^, and overall *F* statistic were used to describe model performance. All tests were two-sided, and *p* < 0.05 was considered statistically significant.

### 2.6 Ethical consideration

Ethical approval for this study was obtained from the Ethics Committee of the Affiliated Hospital of Jiangsu University (Approval No.: KY2025H0717-02). Throughout the research process, the research team adhered to the Declaration of Helsinki to safeguard the rights and interests of all study participants.

## 3 Results

### 3.1 Participants’ demographics

A total of 315 questionnaires were distributed, and 301 of them were valid, with an effective recovery rate of 95.6%. The detailed content of the general information is shown in Table 1.

**Table 1:** General characteristics (*n* = 301).

Projects	Groups	Number of cases (percentage, %)	HPLP-II R (Scores, $\bar{x}\pm S$ )	Test statistic	*p-*value
Gender	Male	142(47.2)	96.15 ± 24.86	−0.443^1^	0.658
	Female	159(52.8)	97.36 ± 22.28		
Age (years)	18–44	99(32.9)	95.54 ± 23.62	3.178^2^	0.043
	45–59	119(39.5)	94.07 ± 22.64		
	≥60	83(27.6)	102.19 ± 23.96		
Place of residence	City	209(69.4)	98.39 ± 22.12	1.783^1^	0.076
	Village	92(30.6)	93.16 ± 26.15		
Marital status	Unmarried	65(21.6)	98.54 ± 24.87	3.495^2^	0.016
	Married	217(72.1)	94.94 ± 22.64		
	Divorced	11(3.7)	108.09 ± 22.17		
	Widowed	8(2.7)	117.13 ± 26.19		
Educational level	Primary school and below	19(6.3)	100.53 ± 31.17	0.704^2^	0.550
	Middle school	47(15.6)	94.23 ± 25.62		
	High school (technical secondary school, vocational college, technical school)	91(30.2)	95.01 ± 24.40		
	University degree or above	144(47.8)	98.26 ± 21.04		
Employment status	In-service	199(66.1)	93.58 ± 22.44	3.153^2^	0.015
	Resigned	6(2.0)	97.0 ± 19.36		
	Retirement	30(10.0)	106.43 ± 23.74		
	Freelance work	45(15.0)	102.84 ± 26.407		
	Student	21(7.0)	100.43 ± 22.71		
Monthly per capita household income (CNY)	<3,000	47(15.6)	107.91 ± 26.11	8.014^2^	< 0.001
	3,000–5,000	112(37.2)	91.94 ± 22.74		
	>5,000	142(47.2)	96.94 ± 22.04		
The role of a sick parent	Father	136(45.2)	97.39 ± 23.89	0.488^2^	0.694
	Mother	127(42.2)	95.39 ± 22.67		
	Father and Mother	38(12.6)	99.32 ± 25.13		
Duration of diabetes (years)	<5	86(28.6)	99.06 ± 21.93	0.705^2^	0.495
	5–10	89(29.6)	94.85 ± 23.84		
	>10	126(41.9)	96.61 ± 24.34		
Awareness of the genetic risk of diabetes	Yes	200(66.4)	99.21 ± 22.39	2.530^1^	0.012
	No	101(33.6)	92.01 ± 25.00		
Living with a parent with type 2 diabetes	Yes	186(61.8)	97.14 ± 23.68	0.327^1^	0.744
	No	115(38.2)	96.23 ± 23.30		
Diabetes-related complications in the parent with type 2 diabetes	Yes	98(32.6)	95.92 ± 22.09	−0.447^1^	0.655
	No	203(67.4)	97.21 ± 24.19		
Type of diabetes treatment received by the parent with type 2 diabetes	Lifestyle intervention	75(24.9)	93.41 ± 25.05	0.728^2^	0.536
	Oral hypoglycemic drugs	116(38.5)	97.53 ± 22.50		
	Insulin injection	46(15.3)	97.63 ± 21.16		
	Oral hypoglycemic drugs + Insulin injection	64(21.3)	98.80 ± 23.50		
Disease risk perception profile	low risk perception profile	129(42.9%)	92.70 ± 26.87	4.584^2^	0.011
	affective-low risk perception profile	91(30.2%)	97.41 ± 22.52		
	affective-elevated risk perception profile	81(26.9%)	102.62 ± 16.83		

^1^ *t*, independent-samples *t*-test; ^2^ *F*, one-way analysis of variance.

Source: Authors’ own.

### 3.2 The perception of disease risk and the score of healthy lifestyle in the offspring of patients with type 2 diabetes

The TRIRISK score of the offspring of type 2 diabetic patients was (56.97 ± 25.61) points. Among them, the deliberative risk perception dimension scored (19.76 ± 9.43) points, the affective risk perception dimension scored (17.63 ± 10.15) points, and the experiential risk perception dimension scored (19.58 ± 8.56) points. The score of the Health Promotion Lifestyle Scale-II R was (96.79 ± 23.50) points, among which the nutrition dimension scored (13.47 ± 4.25) points, the physical exercise dimension scored (18.37 ± 5.11) points, the health responsibility dimension scored (22.67 ± 7.34) points, the interpersonal relationship dimension scored (14.47 ± 3.37) points, the spiritual growth dimension scored (13.75 ± 3.71) points, and the stress management dimension scored (14.24 ± 3.89) points.

### 3.3 The potential profile analysis results of disease risk perception in the offspring of patients with type 2 diabetes

The three TRIRISK dimensions were used as observed indicators in the latent profile analysis, with models containing one to four latent profiles fitted sequentially (Table 2). As the number of profiles increased, the AIC, BIC, and aBIC values generally decreased. Both the LMR-LRT and BLRT were statistically significant for the two- and three-profile models (*p* < 0.05), indicating improved model fit compared to the preceding model. Although the four-profile model yielded lower AIC, BIC, and aBIC values and a significant BLRT (*p* < 0.001), its LMR-LRT was not statistically significant (*p* = 0.601), indicating that the four-profile solution did not provide a statistically significant improvement over the three-profile solution according to this criterion. Additionally, entropy decreased from 0.856 in the three-profile model to 0.835 in the four-profile model. The three-profile solution also produced reasonably balanced profile proportions (42.86%, 30.23%, and 26.91%), with no very small profile, supporting the substantive interpretability of the solution. Therefore, considering model fit indices, classification accuracy, profile sizes, parsimony, and substantive interpretability, the three-profile solution was retained.

**Table 2:** Fitting indices of latent profile model for disease risk perception among offspring of patients with type 2 diabetes mellitus (*n* = 301).

Categories	AIC	BIC	aBIC	Entropy	LMR-LRT (*p* value)	BLRT (*p* value)	Profile proportions
1	2571.598	2593.841	2574.812	–	–	–	1.000
2	2124.777	2161.848	2130.134	0.869	<0.001	<0.001	0.5714/0.4286
3	2015.969	2067.868	2023.468	0.856	0.0008	<0.001	0.4286/0.3023/0.2691
4	1978.560	2045.646	1988.560	0.835	0.6010	<0.001	0.1661/0.4120/0.2691/0.1528

AIC, Akaike information criterion; BIC, Bayesian information criterion; aBIC, sample-size-adjusted Bayesian information criterion; LMR-LRT, Lo–Mendell–Rubin adjusted likelihood ratio test; BLRT, bootstrap likelihood ratio test.

Source: Authors’ own.

The profiles were labeled based on the relative score patterns observed across the three TRIRISK dimensions. Profile 1 was labeled “low risk perception profile” because it exhibited relatively low scores across all three dimensions (*n* = 129, 42.86%). Profile 2 was labeled “affective-low risk perception profile” because it showed moderate-low scores overall, with particularly low affective risk perception (*n* = 91, 30.23%). Profile 3 was labeled “affective-elevated risk perception profile” because it showed moderate scores overall, with relatively higher affective risk perception (*n* = 81, 26.91%), as shown in Figure 1.

**Figure 1:**
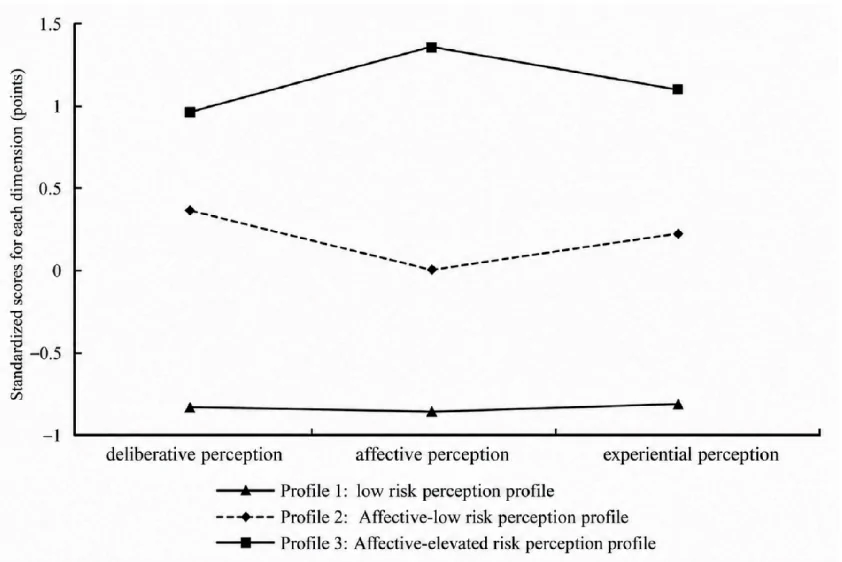
Characteristics of the three latent profiles of disease risk perception among offspring of patients with type 2 diabetes mellitus. Source: Authors’ own.

### 3.4 The results of the univariate analysis of the potential profiles of disease risk perception in the offspring of patients with type 2 diabetes

Significant differences in Health-Promoting Lifestyle Profile-II Revised scores were observed across the three latent profiles of disease risk perception among offspring of patients with type 2 diabetes. Statistically significant variations in HPLP-II R scores were also found in subgroups stratified by age, marital status, employment status, monthly per capita household income (CNY), and awareness of the genetic risk of diabetes. Detailed results are presented in Table 1.

### 3.5 Results of multiple linear regression analysis for health-promoting lifestyles among offspring of patients with type 2 diabetes

Multiple linear regression analysis was conducted with the total HPLP-II R score as the dependent variable. Variables that showed statistically significant differences in the univariate analyses, along with the disease risk perception profiles, were simultaneously entered into the model using the Enter method. The overall regression model was statistically significant (*p* < 0.001). Compared to participants with a monthly per capita household income of less than 3,000 CNY, those with incomes of 3,000–5,000 CNY (*p* = 0.041) and greater than 5,000 CNY (*p* = 0.027) had lower HPLP-II R scores. Compared to Profile 1, participants in Profile 2 (*p* < 0.001) and Profile 3 (*p* < 0.001) had higher HPLP-II R scores. Other variables included in the model were not statistically significant (*p* > 0.05). Detailed regression outputs are presented in Table 3, and the assignment of independent variables is shown in Table 4. Regression diagnostics indicated no serious multicollinearity, with tolerance values ranging from 0.367 to 0.902 and variance inflation factor (VIF) values from 1.109 to 2.727. The Durbin–Watson statistic was 1.519. Standardized residuals ranged from −2.289 to 2.639, with none exceeding an absolute value of 3.

**Table 3:** Multiple linear regression analysis of health-promoting lifestyle among offspring of patients with type 2 diabetes mellitus.

Independent variable	*B*	SE	*β*	*t*	*p*	95% CI for B
Constant	92.239	5.405	–	17.065	<0.001	81.600 to 102.878
Awareness of the genetic risk of diabetes: No vs. Yes	−0.174	3.074	−0.003	−0.057	0.955	−6.225 to 5.877
Age (years)						
45–59 vs. 18–44	4.072	3.762	0.083	1.082	0.280	−3.333 to 11.476
≥60 vs. 18–44	8.011	4.632	0.150	1.729	0.085	−1.107 to 17.129
Marital status						
Unmarried vs. married	4.189	4.229	0.072	0.991	0.323	−4.135 to 12.514
Widowed vs. married	−7.591	8.945	−0.051	−0.849	0.397	−25.197 to 10.016
Divorced vs. married	0.946	7.411	0.007	0.128	0.899	−13.641 to 15.533
Employment status						
Resigned vs. in-service	14.771	10.257	0.086	1.440	0.151	−5.418 to 34.960
Retired vs. in-service	−4.034	5.314	−0.051	−0.759	0.448	−14.493 to 6.424
Freelance vs. in-service	−0.672	4.236	−0.010	−0.159	0.874	−9.009 to 7.665
Student vs. in-service	3.784	6.166	0.040	0.614	0.540	−8.353 to 15.920
Monthly per capita household income (CNY)						
3,000–5,000 vs. <3,000	−8.695	4.239	−0.176	−2.051	**0.041**	−17.038 to −0.352
>5,000 vs. <3,000	−9.686	4.369	−0.202	−2.217	**0.027**	−18.285 to −1.087
Disease risk perception profile						
Profile 2 vs. Profile 1	11.793	3.253	0.227	3.625	**<0.001**	5.390 to 18.196
Profile 3 vs. Profile 1	17.053	3.457	0.317	4.933	**<0.001**	10.249 to 23.857

*R* = 0.355, *R*^2^ = 0.126, adjusted *R*^2^ = 0.083, *F* = 2.952, *p* < 0.001, Durbin–Watson = 1.519. Profile 1: low risk perception profile; Profile 2: affective-low risk perception profile; Profile 3: affective-elevated risk perception profile. Bold values indicate statistically significant differences (*p* < 0.05).

Source: Authors’ own.

**Table 4:** Dummy coding of independent variables entered into the multiple linear regression model.

Independent variable	Dummy variables entered into regression (reference group)
Awareness of the genetic risk of diabetes	1 = No, 0 = Yes (Reference: Yes)
Age (years)	45–59 (1 = 45–59, 0 = other), ≥60 (1 = ≥60, 0 = other) (Reference: 18–44)
Marital status	unmarried (1 = unmarried, 0 = other), divorced (1 = divorced, 0 = other), widowed (1 = widowed, 0 = other) (Reference: married)
Employment status	Resigned (1 = Resigned, 0 = other), retirement (1 = retirement, 0 = other), freelance (1 = freelance work, 0 = other), student (1 = student, 0 = other) (Reference: in-service)
Monthly per capita household income (CNY)	3,000–5,000 (1 = 3,000–5,000, 0 = other), >5,000 (1= > 5,000, 0 = other) (Reference: <3,000)
Disease risk perception profile	Profile 2 (1 = Profile 2, 0 = other), Profile 3 (1 = Profile 3, 0 = other) (Reference: Profile 1)

Profile 1, low risk perception profile; Profile 2, affective-low risk perception profile; Profile 3, affective-elevated risk perception profile.

Source: Authors’ own.

## 4 Discussion

### 4.1 Disease risk perception among offspring of patients with type 2 diabetes mellitus was relatively limited and heterogeneous

The results of the present study showed that the mean score of disease risk perception among offspring patients with type 2 diabetes was (56.97 ± 25.61), suggesting a relatively limited level of perceived diabetes risk in this sample. This finding suggests that this high-risk group lacks sufficient vigilance and attention toward their personal disease risk, which is consistent with the research conducted by Liang Fang et al. (17). The existing literature has adopted diverse assessment tools for risk perception, making direct horizontal comparisons of relevant research results difficult. Nevertheless, synthesized evidence indicates that the overall level of disease risk perception among participants in this study is relatively low, which is consistent with the cognitive characteristics of most populations at high risk of chronic diseases.

Although the effectiveness of lifestyle-centered health management has been widely recognized, enormous challenges remain in translating relevant theories into practical interventions targeted at high-risk populations. The State Council issued the Outline of the Healthy China 2030 Plan, which for the first time put forward the concept of proactive health (18). This concept emphasizes the dominant role of individuals in health management, and encourages people to actively engage in the prevention, treatment and rehabilitation of diseases via positive behavioral and lifestyle modifications (19). Nevertheless, the key to stimulating individual health initiative lies in accurate self-perception of health risks and the subsequent intention of behavioral change.

However, existing findings also indicate that disease risk perception in this population cannot be adequately captured by a single overall level alone. Latent profile analysis identified three distinct risk perception patterns: the low risk perception profile (42.86%), the affective-low risk perception profile (30.23%), and the affective-elevated risk perception profile (26.91%). These patterns suggest that adult children of patients with type 2 diabetes exhibit significant heterogeneity in the rational, affective, and experiential dimensions of diabetes risk perception. This observed heterogeneity is reflected not only in differences in the overall level of risk perception but also in the structure of rational, affective, and experiential risk perception. Recognizing this heterogeneity can help healthcare providers move beyond a one-size-fits-all approach to risk communication and health education. In future practice, greater attention should be paid to differences in risk perception patterns across subgroups, and preventive support measures should be tailored to individuals’ cognitive characteristics and actual needs. If confirmed in prospective and intervention studies, such a pattern-based strategy may provide a foundation for more differentiated, population-specific health services.

### 4.2 Disease risk perception profiles were associated with health-promoting behaviors

The results of this study showed that participants in both the affective-low risk perception profile and the affective-elevated risk perception profile had significantly higher levels of health-promoting behaviors than those in the low risk perception profile. This finding suggests that different patterns of disease risk perception are associated with different levels of health-promoting behavior among adult offspring of patients with type 2 diabetes mellitus. The Protection Motivation Theory posits (20) that individuals with higher risk perception are more likely to adopt health-promoting behaviors. Previous studies have also reported associations between illness perception, self-management, and health-promoting behaviors among patients with diabetes and other chronic diseases (21–23). These findings provide further support for the relevance of risk-related perceptions to health behavior, although the underlying mechanisms may differ across populations. Among the three profiles, participants in the low risk perception profile showed relatively low levels across all three TRIRISK dimensions and had the lowest level of health-promoting behaviors. This pattern suggests that consistently low perceived risk may be accompanied by less engagement in preventive health behaviors. For this subgroup, strengthening awareness of personal diabetes risk and the potential benefits of early prevention may therefore be particularly important. Risk visualization, individualized risk feedback, and family-supported health education could be explored as possible approaches to enhance risk awareness and encourage preventive action. Participants in the affective-low risk perception profile showed relatively lower affective risk perception but still had significantly higher health-promoting behavior scores than those in the low risk perception profile. This finding suggests that health-promoting behaviors may not depend on the affective component of risk perception alone, but rather on the combined pattern of deliberative, affective, and experiential risk perception. For this subgroup, health communication may need to go beyond the simple provision of disease knowledge and pay greater attention to helping individuals connect risk information with personally meaningful health goals and preventive actions. Participants in the affective-elevated risk perception profile also had significantly higher levels of health-promoting behaviors than those in the low risk perception profile. Their relatively higher affective risk perception suggests that emotional responses may represent an important component of how some individuals perceive diabetes risk. For this subgroup, future health education could consider maintaining appropriate risk awareness while providing clear, structured, and actionable behavioral guidance to support sustained health management. Taken together, these findings suggest that the relationship between disease risk perception and health-promoting behaviors may be better understood from a multidimensional profile perspective rather than from any single risk-perception component in isolation. Recognition of these distinct patterns may help healthcare providers move beyond a one-size-fits-all approach to diabetes risk communication and health education. Evidence from other high-risk populations suggests that risk assessment combined with lifestyle guidance may support healthier behaviors (24). If confirmed in prospective and intervention studies, profile-informed strategies could provide a basis for more differentiated preventive support for adult offspring of patients with type 2 diabetes mellitus.

### 4.3 An unexpected association between household income and health-promoting behaviors

The social economic status is closely related to health outcomes and health-related behaviors. People with a lower social economic status often encounter greater obstacles in accessing medical resources and maintaining a healthy lifestyle (25–27). However, in this study, after adjusting for other variables using regression models, participants with a monthly per capita household income of 3,000–5,000 yuan and those above 5,000 yuan had lower HPLP-II R scores than those with an income below 3,000 yuan. This finding differs from the generally reported correlation that higher social economic resources lead to better health behaviors (28). It is notable that the pattern observed in this study does not show a monotonic trend across different income categories, suggesting that the relationship between family income and health-promoting behaviors may be more complex than a simple linear gradient. One possible explanation is that social economic resources do not necessarily directly translate into healthier lifestyles, as health behaviors may also be influenced by occupational requirements, health literacy, lifestyle patterns, and personal health priorities. These factors may vary among different income groups, which may help to explain the observed association. Therefore, the results of this study may suggest that family income may be a contextual factor in the broader context of behaviors and the social economic environment, rather than an independent determinant of health-promoting behaviors. This unexpected pattern requires further research in more representative populations to clarify how social economy and behavioral factors interact and jointly shape healthy-lifestyle-promoting behaviors.

### 4.4 Limitations

This study has several limitations. First, participants were recruited using convenience sampling from the outpatient and inpatient departments of a single tertiary hospital. Adult offspring who accompanied their parents to hospital visits may differ from those who did not accompany their parents, particularly in terms of family health involvement and health awareness. Therefore, selection bias cannot be excluded, and the generalizability of the findings to the broader population of adult offspring of individuals with type 2 diabetes mellitus should be interpreted with caution. Second, the cross-sectional design precludes determination of temporal or causal relationships between disease risk perception and health-promoting behaviors. Third, the study variables were mainly assessed using self-reported questionnaires and may therefore be affected by recall or social desirability bias. Future multicenter prospective studies involving more diverse populations are needed to validate the identified risk-perception profiles and further clarify their longitudinal associations with health behaviors.

## 5 Conclusion

This study identified three distinct latent profiles of disease risk perception among adult offspring of patients with type 2 diabetes mellitus, and these profiles were significantly associated with health-promoting behaviors. These findings highlight the heterogeneity of risk perception within this high-risk population and suggest that disease risk perception may be a useful basis for developing more differentiated and person-centered diabetes prevention strategies. In future practice, assessment of risk-perception profiles could be incorporated into routine risk communication and health education to better match preventive support with individual needs. Further prospective and intervention studies are warranted to determine whether profile-based prevention strategies can promote sustained health behavior changes and contribute to delaying the development of type 2 diabetes.

## Data Availability

The original contributions presented in the study are included in the article/supplementary material, further inquiries can be directed to the corresponding authors.
